# Hippocampal atrophy is associated with hearing loss in cognitively normal adults

**DOI:** 10.3389/fnins.2023.1276883

**Published:** 2023-10-24

**Authors:** Ye Ji Shim, Wi Hoon Jung, Alexander J. Billig, William Sedley, Jae-Jin Song

**Affiliations:** ^1^Department of Otorhinolaryngology-Head and Neck Surgery, Healthcare System Gangnam Center, Seoul National University Hospital, Seoul, Republic of Korea; ^2^Sensory Organ Research Institute, Seoul National University Medical Research Center, Seoul, Republic of Korea; ^3^Department of Psychology, Gachon University, Seongnam, Republic of Korea; ^4^UCL Ear Institute, University College London, London, United Kingdom; ^5^Translational and Clinical Research Institute, Newcastle University Medical School, Newcastle upon Tyne, United Kingdom; ^6^Department of Otorhinolaryngology-Head and Neck Surgery, Seoul National University Bundang Hospital, Seongnam, Republic of Korea

**Keywords:** hippocampus, hearing loss, atrophy, aging, structural neuroimaging

## Abstract

**Objectives:**

A growing body of evidence suggests that age-related hearing loss (HL) is associated with morphological changes of the cerebral cortex, but the results have been drawn from a small amount of data in most studies. The aim of this study is to investigate the correlation between HL and gray matter volume (GMV) in a large number of subjects, strictly controlling for an extensive set of possible biases.

**Methods:**

Medical records of 576 subjects who underwent pure tone audiometry, brain magnetic resonance imaging (MRI), and the Korean Mini-Mental State Exam (K-MMSE) were reviewed. Among them, subjects with normal cognitive function and free of central nervous system disorders or coronary artery disease were included. Outliers were excluded after a sample homogeneity check. In the end, 405 subjects were enrolled. Pure tone hearing thresholds were determined at 0.5, 1, 2, and 4 kHz in the better ear. Enrolled subjects were divided into 3 groups according to pure tone average: normal hearing (NH), mild HL (MHL), and moderate-to-severe HL (MSHL) groups. Using voxel-based morphometry, we evaluated GMV changes that may be associated with HL. Sex, age, total intracranial volume, type of MRI scanner, education level, K-MMSE score, smoking status, and presence of hypertension, diabetes mellitus and dyslipidemia were used as covariates.

**Results:**

A statistically significant negative correlation between the hearing thresholds and GMV of the hippocampus was elucidated. Additionally, in group comparisons, the left hippocampal GMV of the MSHL group was significantly smaller than that of the NH and MHL groups.

**Conclusion:**

Based on the negative correlation between hearing thresholds and hippocampal GMV in cognitively normal old adults, the current study indicates that peripheral deafferentation could be a potential contributing factor to hippocampal atrophy.

## 1. Introduction

Age-related hearing loss (ARHL) is one of the most common sensory impairments in elderly subjects. ARHL is characterized by difficulties in speech understanding in acoustically adverse settings, delayed central auditory processing, and compromised sound localization (Gates and Mills, 2005). Due to the high prevalence and its impact on everyday life, ARHL is a great social burden. In subjects over 65 years of age, one in three suffer from hearing loss (HL), and in those over 80, HL affects more than a half (Roth et al., 2011; Homans et al., 2017). ARHL is the leading cause of years lived with disability for population over 70 years of age (Collaborators, 2021). Due to population growth and aging, the number of people living with HL is expected to rise by 56.1%, from 1.57 billion in 2019 to 2.45 billion in 2050 (Collaborators, 2021). Therefore, the social burden of ARHL is expected to rise continuously.

In addition to the high prevalence of presbycusis, ARHL has received considerable attention associated with cognitive decline. An expanding array of research indicates a correlation between ARHL and both cognitive deterioration and an increased risk for dementia (Lin et al., 2011, 2013; Deal et al., 2017). Furthermore, the Lancet Commission on Dementia Prevention identified ARHL as the most significant modifiable risk factor for dementia (Livingston et al., 2017).

Although the relationship between ARHL and cognitive decline has been confirmed in many epidemiological studies, how the two are linked remains unclear. Recently, many studies have attempted to elucidate the mechanism, including through analysis of brain imaging data. Structural imaging studies have been conducted to identify the association between HL and brain volumes of auditory- and non-auditory regions of the brain. However, there are stark disparities among studies. The auditory cortex has been the most frequently investigated region, but even the volumetric changes in the auditory cortex have shown inconsistent results among studies (Lin et al., 2014; Giroud et al., 2017; Rigters et al., 2017; Ren et al., 2018; Eckert et al., 2019). Also, other studies have evaluated the association between HL and a more extensive set of regions known to support auditory perception, such as the cingulate cortex, amygdala, and hippocampus, but the relationship between HL and cortical reorganization has not been fully elucidated yet (Uchida et al., 2018; Armstrong et al., 2019; Belkhiria et al., 2019, 2020).

In this regard, we sought to investigate the correlation between volumetric changes of the cerebral cortex and ARHL using whole-brain exploratory analysis in a large series of subjects. Also, by controlling numerous variables that can affect brain volume, the HL-related changes in the cerebral cortex in the current study were minimally biased by other factors.

## 2. Materials and methods

### 2.1. Subjects

This is a single-center retrospective cohort study. We retrospectively reviewed subjects who visited Seoul National University Hospital Healthcare System Gangnam Center from January 2017 to June 2020. A total of 576 adults who were older than 50 years and had undergone pure tone audiometry, brain magnetic resonance imaging (MRI), and the Korean Mini-Mental State Exam (K-MMSE) were initially screened. Subjects with cognitive decline (K-MMSE score < 24) (*n* = 101), central nervous system (CNS) disorders (*n* = 13), coronary artery disease (CAD) requiring percutaneous coronary intervention or coronary artery bypass grafting (*n* = 51) were excluded from the study, along with outliers from a voxel-based morphometry (VBM) sample homogeneity test (*n* = 10) (Kang et al., 1997). Four subjects had both CNS disorder and CAD. As a result, 405 eligible subjects were finally enrolled in this study. The pure tone audiometric thresholds were calculated by averaging thresholds at 0.5, 1, 2, and 4 kHz of the better ear. Enrolled subjects were divided into three groups according to pure tone average based on the WHO’s grades of hearing impairment: normal hearing (NH, <25 dB), mild hearing loss (MHL, ≥25 dB and <40 dB), and moderate-to-severe hearing loss (MSHL, ≥40 dB) (World Health Organization, 1991). The study was approved by the Institutional Review Board of the Clinical Research Institute at Seoul National University Hospital (IRB No. 2009-011-1154).

### 2.2. Image acquisition

The subjects underwent brain structural MRI using one of three types of scanner: 1.5 T brain MRI (i) Achieva (179 subjects; Philips Medical Systems, Best, The Netherlands), (ii) MAGNETOM Espree (105 subjects; Siemens, Erlangen, Germany) or 3 T brain MRI (iii) MAGNETOM Skyra (121 subjects; Siemens, Erlangen, Germany). Structural images were acquired with (i) 3D T1 weighted TFE sequence with repetition time (TR) = 7.5 ms, echo time (TE) = 3.4 s, flip angle (FA) = 8°, voxel size = 1.0 × 1.0 × 1.0 ${\mathrm{mm}}^{3}$ , slice thickness = 1 mm, 160 sagittal slices, field of view (FOV) = 240 mm, (ii) 3D T1 weighted TFL sequence with TR = 1,020 ms, TE = 4.29 s, FA = 15°, voxel size = 0.9 × 0.9 × 1.0 ${\mathrm{mm}}^{3}$ , slice thickness = 1 mm, 160 sagittal slices, FOV = 240 mm, or (iii) 3D T1 weighted MPRAGE sequence with TR = 1,600 ms, TE = 2.84 s, FA = 9°, voxel size = 0.5 × 0.5 × 1.0 ${\mathrm{mm}}^{3}$ , slice thickness = 1 mm, 192 sagittal slices, FOV = 240 mm.

### 2.3. Preprocessing of MRI images for VBM analysis

VBM analysis of the T1-weighted images was performed using the Computational Anatomy Toolbox (CAT12^1^) implemented in Statistical Parametric Mapping 12 (SPM12^2^) software using MATLAB (R2020b). For pre-processing, default settings according to the standard protocol were used. All scans were segmented into gray matter (GM), white matter, and cerebrospinal fluid and spatially normalized to Montreal Neurological Institute (MNI) stereotactic space using DARTEL (diffeomorphic anatomical registration through an exponentiated Lie algebra) algorithm. Then, normalized GM images were modulated (i.e., scaled by the amount of expansions and contractions that has occured during spatial normalization), so that voxel-wise gray matter volumes (GMV) remains the same as in individual native space. After segmentation, a quality check was conducted. The normalized segmented images were smoothed using an 8-mm FWMH Gaussian kernel. We used an absolute masking threshold of 0.1.

### 2.4. Statistical analysis

Multiple regression analysis was performed to evaluate correlations between hearing thresholds and GMV. Sex, age, total intracranial volume (TIV), type of MRI scanner, education level, MMSE score, hypertension, diabetes mellitus, dyslipidemia, and smoking status were used as covariates to minimize bias while evaluating correlations between hearing thresholds and GMV. The differences in GMV between the three groups were evaluated with factorial analysis with the type of MRI scanner and group according to PTA as factors. By analyzing the data in this way, inter-scanner differences were adjusted for as described in previous literature (Pardoe et al., 2008). Again, sex, age, TIV, education level, MMSE score, hypertension, diabetes mellitus, dyslipidemia, and smoking status were used as covariates. The *post-hoc* test was performed using the Mann–Whitney U test with Bonferroni correction. All results were corrected for multiple comparisons to a significant level of voxel-level *p* < 0.05, family-wise error (FWE)-corrected, and a cluster size >10 voxels was adopted. Because all continuous clinical and demographic data (better ear hearing level, TIV, and MMSE) were not normally distributed (confirmed by the Shapiro-Wilks test, *p* < 0.05 for all continuous parameters), we compared the three groups using the Kruskal-Wallis test for the continuous data and the χ^2^-test for the categorical data. All statistical analyses were performed with the SPSS 23 (IBM Corporation, New York, USA) package using a two-sided test with a significance level of 0.05.

### 2.5. Covariates

In statistical analysis, 10 covariates (sex, age, TIV, type of MRI scanner, education level, K-MMSE score, hypertension, diabetes mellitus, dyslipidemia, and smoking status) were adjusted. Variables that could potentially be confounders in the relationship between hearing thresholds and brain volume, and variables known to be associated with HL were used as covariates. Smoking status was based on self-report. The diagnosis of hypertension, diabetes mellitus, and dyslipidemia was established based on a history of a physician diagnosis, pharmacologic treatment, and self-report.

## 3. Results

### 3.1. Demographic and clinical characteristics

The demographic and clinical characteristics of 405 subjects are summarized in Table 1. Two hundred and forty-two subjects with a PTA < 25 dB were classified as the NH group, 91 subjects with a PTA ≥ 25 dB and < 40 dB were classified as the MHL group, and 72 subjects with a PTA ≥ 40 dB were classified as the MSHL group. There were no significant differences between the three groups with regard to sex, TIV, education level, dyslipidemia, and smoking status. The age of the NH group was significantly lower than that of the MHL and the MSHL groups. The K-MMSE score of the NH group was significantly higher than that of the MHL and the MSHL groups. The prevalence of hypertension and diabetes was higher in the MSHL group than in the NH group.

**Table 1:** Clinical and demographic characteristics of the three groups.

	Normal (*n* = 242)	Mild HL (*n* = 91)	Mod to severe HL (*n* = 72)	*p-*value
Age, mean (SD), years	65.09 (6.92)	70.79 (6.93)	73 (6.81)	<0.001*
Sex, *n* (%)				0.091
Male	96 (39.7)	38 (41.8)	39 (54.2)	
Female	146 (60.3)	53 (58.2)	33 (45.8)	
TIV, mean (SD), ${\mathrm{cm}}^{3}$	1433.20 (144.05)	1437.93 (140.52)	1446.26 (145.24)	0.620
Education, mean (SD), years	14.15 (2.74)	14.02 (3.57)	13.73 (3.52)	0.778
MMSE, mean (SD)	27.22 (2.40)	26.64 (1.69)	26.45 (1.84)	<0.001*
PTA, mean (SD), dB	16.85 (4.79)	31.19 (4.19)	49.89 (9.17)	<0.001*
Hypertension, *n* (%)	84 (34.7)	41 (45.1)	39 (54.2)	<0.008*
Diabetes Mellitus, *n* (%)	39 (16.1)	17 (18.7)	21 (29.2)	<0.047*
Dyslipidemia, *n* (%)	111 (45.9)	50 (54.9)	36 (50.0)	0.326
Smoking, *n* (%)				0.131
Never-smoker	189 (78.1)	72 (79.1)	49 (68.1)	
Ex-smoker	17 (7.0)	7 (7.7)	5 (6.9)	
Current-smoker	36 (14.9)	12 (13.2)	18 (25)	

Mod, moderate; HL, hearing loss; SD, standard deviation; n, number; TIV, total intracranial volume; MMSE, mini-mental state examination; PTA, pure tone audiometry.

### 3.2. Correlation analyses between GMV and HL

**Figure 1:**
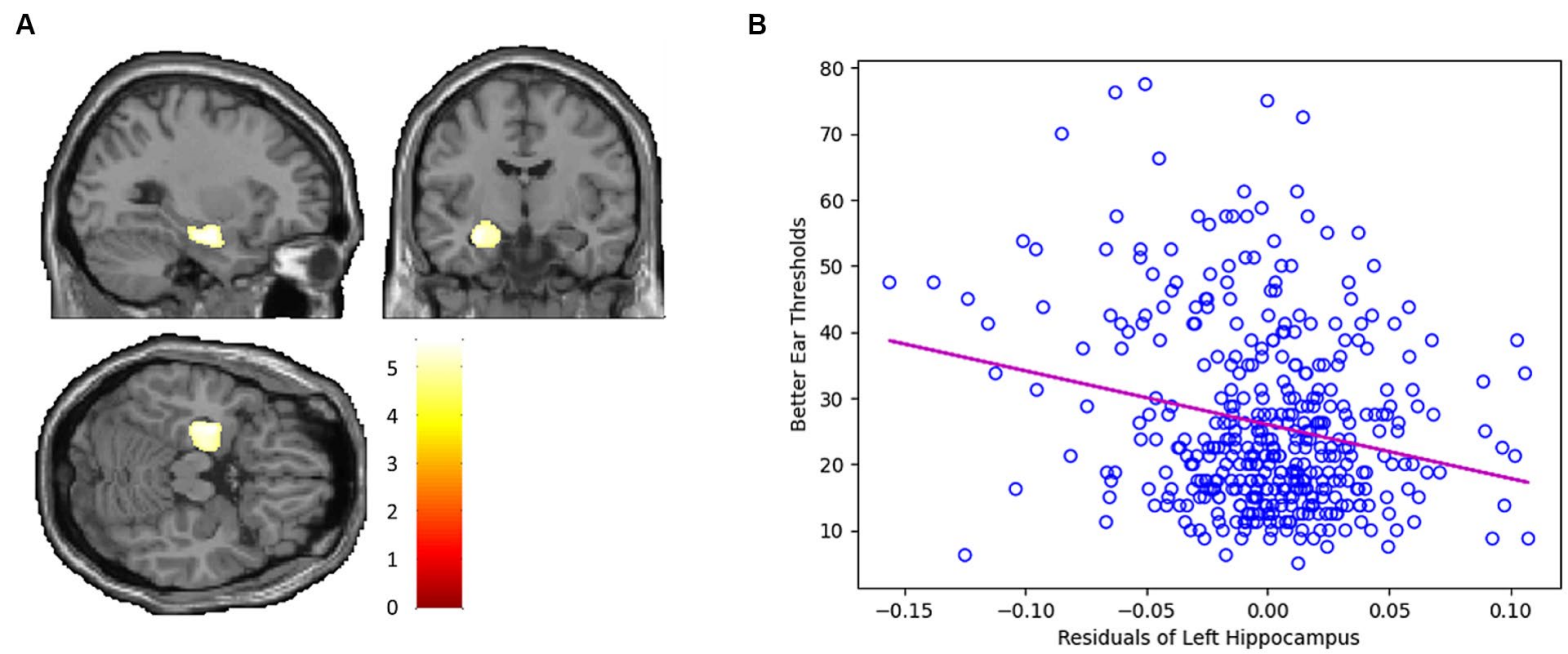
**(A)** Whole-brain voxel-level linear correlation analysis indicates that the volume of the left hippocampus (peak MNI x, y, z coordinates = −29, −9, −18; peak *z*-value = 5.43) is negatively associated with hearing threshold. **(B)** Scatter plot depicting the negative linear correlation between the GMV of the left hippocampus and hearing threshold.

Whole-brain voxel-level linear correlation analyses revealed that the volume of the left hippocampus (peak MNI x, y, z coordinates = −29, −9, −18; peak *z*-value = 5.43) was negatively associated with hearing threshold (Figure 1).

### 3.3. Group comparison of GMV

**Figure 2:**
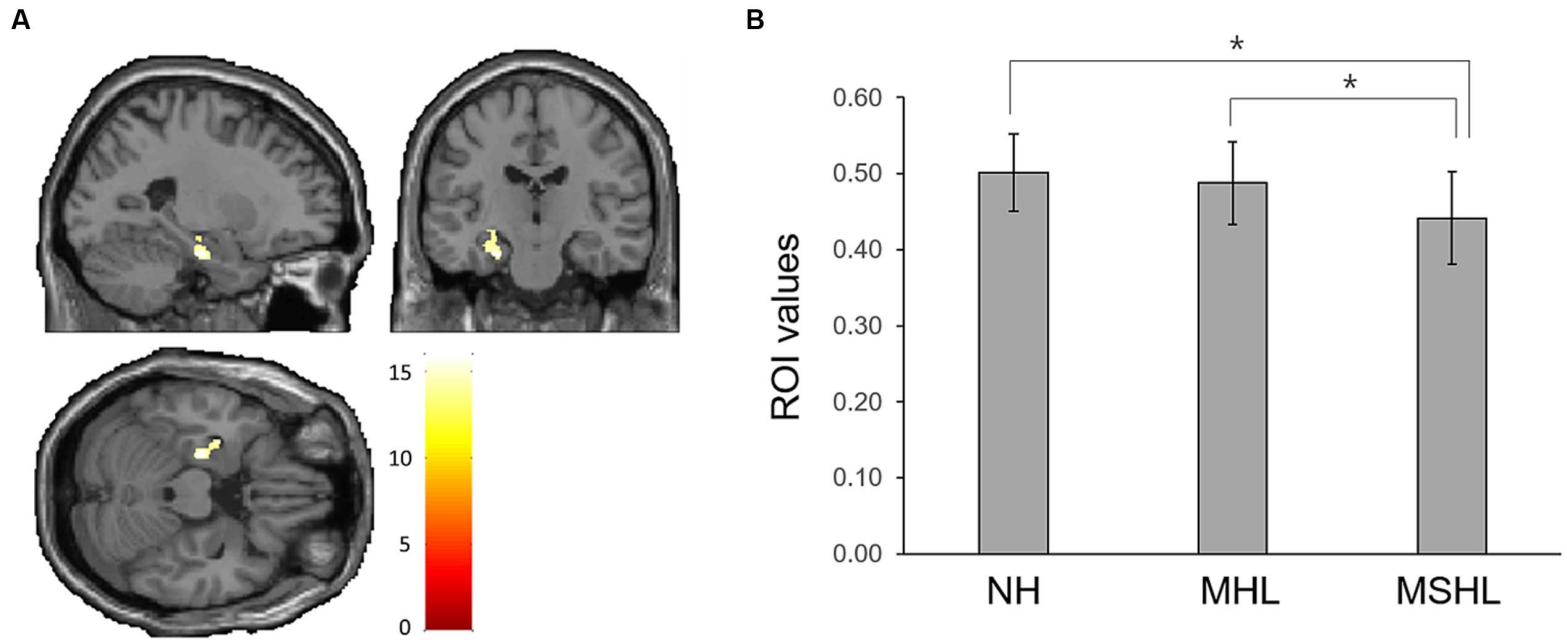
**(A)** The left hippocampus (peak MNI x, y, z coordinates = −24, −18, −23; peak *z*-value = 5.06) showed a significant difference between the groups. **(B)** On *post-hoc* analysis, the moderate to severe hearing loss group showed significantly decreased left hippocampal volume compared to that of the mild hearing loss (MHL) (*p* < 0.001) and the normal hearing (NH) groups (*p* < 0.001). There were no significant differences between the MHL group and the NH group. Asterisks (*) indicate a statistically significant difference with a *p*-value of less than 0.05.

The factorial analysis found that the left hippocampus (peak MNI x, y, z coordinates = −24, −18, −23; peak *z*-value = 5.06) showed a significant difference between the groups (Figure 2A). On *post-hoc* analysis, the MSHL group showed significantly decreased left hippocampal volume compared to that of the MHL (*p* < 0.001) and the NH groups (*p* < 0.001). There were no significant differences between the MHL group and the NH group (Figure 2B).

## 4. Discussion

While evidence suggests that peripheral HL is associated with accelerated cognitive decline and elevated dementia risk (Lin et al., 2011, 2013; Deal et al., 2017) the underlying mechanism remains unclear. As part of elucidating this mechanism, the present study observed an inverse relationship between hearing thresholds and hippocampal GMV, a structure critically involved in dementia development, even among subjects with normal cognitive function.

Consistent with our study, two recent investigations, including a cohort study, have reported associations between HL and diminished hippocampal volume as well as accelerated volume decline (Uchida et al., 2018; Armstrong et al., 2019). Moreover, hippocampal atrophy is an indicator of neurodegeneration, as a proxy of neuronal loss, and is closely tied to accelerated volume reduction in both adults experiencing cognitive decline and those diagnosed with Alzheimer’s Disease (AD) (Jack et al., 2002; de Leon et al., 2004; Shi et al., 2009; Del Sole et al., 2016). Importantly, hippocampal atrophy may occur even before the onset of clinical AD symptoms (Lewczuk et al., 2015; McConathy and Sheline, 2015). Given these considerations, it is plausible that the effect of HL on cognitive decline might be mediated through changes in the hippocampus.

The hippocampus plays a multifaceted role in information processing and memory, with the right hippocampus generally implicated in spatial navigation and visual memory, while the left hippocampus predominantly engages in perception, learning and recall of speech (Ezzati et al., 2016; Billig et al., 2022). Our results specifically implicate the left hippocampus, consistent with its known lateralization. The hippocampus certainly receives and operates on auditory information (for a review, see Billig et al., 2022), and its pyramidal place cells (O’Keefe and Dostrovsky, 1971) can map out dimensions of sound as they do for space, at least in rodents (Aronov et al., 2017). The hippocampus is also active during auditory working memory (Bishop and Miller, 2009; Davis et al., 2011; Kumar et al., 2016; Lad et al., 2020; Kumar et al., 2021) and may provide predictive information to superior temporal sites during continuous speech listening (Michelmann et al., 2021). Given the challenges in speech recognition under acoustically challenging conditions associated with ARHL (Tun et al., 2002; Best et al., 2010), coupled with the left hippocampal functions, it could be hypothesized that the cognitive load on the left hippocampus may be elevated in individuals with ARHL. It has been suggested that excessive activation of hippocampal neurons could lead to hippocampal degeneration and a worsening of AD pathology (de Haan et al., 2012; Beckmann et al., 2020). In this context, Griffiths et al. emphasized the possibility of a bidirectional relationship between medial temporal lobe dysfunction and HL, which could interact with AD pathology (Griffiths et al., 2020). Such a relationship is suggested to be mediated by the decline in spectral and temporal resolution in ARHL that complicates speech-in-noise analysis, thereby necessitating heightened hippocampal activity (Gordon-Salant et al., 2007; Pichora-Fuller et al., 2007). This theory might explain the findings of the present study and the previous two studies (de Haan et al., 2012; Beckmann et al., 2020).

Alternatively, impaired adult hippocampal neurogenesis (AHN) could serve as an explanatory factor for the inverse association between hearing thresholds and hippocampal GMV. The hippocampus is one of the few areas of the brain where neurogenesis persists even during adulthood (Ernst and Frisen, 2015). Recent literature suggests that compromised AHN may contribute to hippocampal atrophy (Anand and Dhikav, 2012; Schloesser et al., 2014; Schoenfeld et al., 2017). Compromised AHN is observed in the early stages of AD, even before clinical symptoms and amyloid deposition occur (Lazarov and Hollands, 2016; Liu et al., 2021). Multiple AD pathology-related factors, including tau protein and Aβ, among others, disturb AHN and exacerbate cognitive deficits (Mu and Gage, 2011; Hollands et al., 2017; Liu et al., 2021). In animal studies, noise-induced hearing loss (NIHL) has been shown to decrease AHN and impair memory (Kraus et al., 2010;Liu et al., 2016; Shukla et al., 2019), with these effects persisting up to 12 months after noise exposure evidenced by increased hippocampal tau-phosphorylation, which may inhibit AHN (Pristera et al., 2013; Komuro et al., 2015; Park et al., 2018; Houben et al., 2019). Since NIHL is a prominent type of acquired HL and shares pathophysiological mechanisms with ARHL, the findings from NIHL studies could be applied to ARHL (Yang et al., 2015; Hederstierna and Rosenhall, 2016; Liberman, 2017). Notably, the cited animal studies propose that HL, rather than noise trauma *per se*, modulates neurogenesis (Liu et al., 2016; Shukla et al., 2019). This assertion is further corroborated by a study using a conductive hearing loss (CHL) mouse model, which also demonstrated a reduction in AHN following CHL (Kurioka et al., 2021). Recent evidence indicates that neurogenesis-related pathways including cellular differentiation and morphogenesis during development, among others, are associated with hippocampal atrophy (Horgusluoglu-Moloch et al., 2019). Accordingly, the resultant smaller hippocampal volume could be a consequence of HL-modulated AHN. In addition, various factors such as environmental enrichment, exercise, stress, and social isolation affect AHN (Gould et al., 1997; Kempermann et al., 1997; Lemaire et al., 2000; van Praag et al., 2000; Kreisel et al., 2014; Voss et al., 2019), suggesting that HL-induced stress and social isolation could indirectly contribute to AHN and hippocampal atrophy.

In the group comparison of our study, the left hippocampal GMV of the MSHL group was smaller than that of the NH and MHL groups. The MHL group showed no significant difference from the NH group in hippocampal volume. The risk of incident dementia is increased more steeply with moderate to severe HL (Lin et al., 2011; Deal et al., 2017). Moderate to severe HL seems to have a more significant effect on hippocampal atrophy and cognitive function deterioration. Hearing aid use slows the decline of episodic memory, one of the functions of the left hippocampus (Maharani et al., 2018). Early intervention before moderate HL occurs might help prevent the progression of cognitive impairment. Additional research is required to assess the impact of early intervention on cognitive outcomes.

Studies on the association between HL and structural changes of the auditory cortex showed inconsistent results. Our results did not show any correlation between GMV of the auditory cortex and hearing thresholds. This aligns with prior research indicating that the effect of ARHL on the auditory cortex is minimal, while the process of aging exerts a substantial impact (Profant et al., 2013, 2014; Ouda et al., 2015; Profant et al., 2015, 2020). Conversely, some studies have shown a correlation between peripheral HL and GM atrophy in the primary auditory cortex (Peelle et al., 2011; Eckert et al., 2012; Lin et al., 2014; Ren et al., 2018; Uchida et al., 2018). ARHL is a term with a broad scope, and there are various subgroups within it according to the type or etiology of HL. The inclusion in these studies of patients with various types of HL without distinction might result in these inconsistent results.

There are some limitations in our study design. First, since this is a cross-sectional study, while a relationship between HL and hippocampus atrophy is revealed, a causal link between the two is yet to be unveiled. However, while the results are correlational, and therefore do not directly demonstrate a causal relationship, we believe that the best interpretation is that hippocampal atrophy is a consequence of moderate-to-severe HL; this would be consistent with existing evidence for HL causing impaired neurogenesis in hippocampus, as its eventual consequence, though could be the result of alternative structure–function relationships. Second, MRI obtained using scanners of different field strengths were included in this study. In case of a consistent bias, it might be viable to adjust the scanner type to enhance cross-site comparability. In our study, the scanner type was adjusted as a covariate in correlation analysis. In addition, to check the reliability of these results, a comparison between groups was performed using factorial analysis, as introduced by Pardoe et al. to correct inter-scanner differences in multi-site studies (Pardoe et al., 2008). The factorial analysis also confirmed the association between HL and hippocampus atrophy.

The strengths of our study lie in its rigorous design and the use of a large sample size (*n* = 405). A key advantage is our meticulous approach to address potential biases and confounding factors. By carefully controlling for covariates such as age, sex, total intracranial volume, education level, cognitive status, hypertension, diabetes mellitus, dyslipidemia, and smoking status in our statistical analysis, we ensure robustness and validity of our findings. The substantial sample size further enhanced the study’s statistical power, allowing for a comprehensive and reliable investigation of the association between HL and brain structure, leading to more meaningful conclusions.

## 5. Conclusion

This study demonstrates a negative correlation between hearing thresholds and hippocampal GMV in cognitively normal old adults. This result suggested that peripheral deafferentation could be a potential contributing factor to hippocampal atrophy.

## Data availability statement

The original contributions presented in the study are included in the article/supplementary material, further inquiries can be directed to the corresponding author.

## Ethics statement

The studies involving humans were approved by Institutional Review Board of the Clinical Research Institute at Seoul National University Hospital (IRB No. 2009-011-1154). The studies were conducted in accordance with the local legislation and institutional requirements. Written informed consent for participation was not required from the participants or the participants’ legal guardians/next of kin in accordance with the national legislation and institutional requirements.

## Author contributions

YS: Data curation, Formal analysis, Funding acquisition, Investigation, Methodology, Visualization, Writing – original draft, Writing – review & editing. WJ: Data curation, Formal analysis, Investigation, Methodology, Supervision, Validation, Visualization, Writing – original draft, Writing – review & editing. AB: Investigation, Writing – review & editing. WS: Investigation, Writing – review & editing. J-JS: Conceptualization, Data curation, Formal analysis, Investigation, Methodology, Supervision, Writing – original draft, Writing – review & editing.

## Footnotes

^1^http://www.neuro.uni-jena.de/cat/

^2^http://www.fil.ion.ucl.ac.uk/spm/

## References

[ref1] Anand K. S. Dhikav V. (2012). Hippocampus in health and disease: An overview. Ann. Indian Acad. Neurol. 15, 239–246. doi: 10.4103/0972-2327.104323 23349586 PMC3548359

[ref2] Armstrong N. M. An Y. Doshi J. Erus G. Ferrucci L. Davatzikos C.. (2019). Association of midlife hearing impairment with late-life temporal lobe volume loss. JAMA Otolaryngol. Head Neck Surg. 145, 794–802. doi: 10.1001/jamaoto.2019.1610 31268512 PMC6613307

[ref3] Aronov D. Nevers R. Tank D. W. (2017). Mapping of a non-spatial dimension by the hippocampal-entorhinal circuit. Nature 543, 719–722. doi: 10.1038/nature21692 28358077 PMC5492514

[ref4] Beckmann D. Feldmann M. Shchyglo O. Manahan-Vaughan D. (2020). Hippocampal synaptic plasticity, spatial memory, and neurotransmitter receptor expression are profoundly altered by gradual loss of hearing ability. Cereb. Cortex 30, 4581–4596. doi: 10.1093/cercor/bhaa061 32202614 PMC7325716

[ref5] Belkhiria C. Vergara R. C. San Martin S. Leiva A. Marcenaro B. Martinez M.. (2019). Cingulate cortex atrophy is associated with hearing loss in presbycusis with cochlear amplifier dysfunction. Front. Aging Neurosci. 11:97. doi: 10.3389/fnagi.2019.00097 31080411 PMC6497796

[ref6] Belkhiria C. Vergara R. C. San Martin S. Leiva A. Martinez M. Marcenaro B.. (2020). Insula and amygdala atrophy are associated with functional impairment in subjects with presbycusis. Front. Aging Neurosci. 12:102. doi: 10.3389/fnagi.2020.00102 32410980 PMC7198897

[ref7] Best V. Gallun F. J. Mason C. R. Kidd G. Jr. Shinn-Cunningham B. G. (2010). The impact of noise and hearing loss on the processing of simultaneous sentences. Ear Hear. 31, 213–220. doi: 10.1097/AUD.0b013e3181c34ba6 20075737 PMC2836417

[ref8] Billig A. J. Lad M. Sedley W. Griffiths T. D. (2022). The hearing hippocampus. Prog. Neurobiol. 218:102326. doi: 10.1016/j.pneurobio.2022.102326 35870677 PMC10510040

[ref9] Bishop C. W. Miller L. M. (2009). A multisensory cortical network for understanding speech in noise. J. Cogn. Neurosci. 21, 1790–1805. doi: 10.1162/jocn.2009.21118 18823249 PMC2833290

[ref10] Collaborators G. B. D. H. L. (2021). Hearing loss prevalence and years lived with disability, 1990-2019: findings from the Global Burden of Disease Study 2019. Lancet 397, 996–1009. doi: 10.1016/S0140-6736(21)00516-X 33714390 PMC7960691

[ref11] Davis M. H. Ford M. A. Kherif F. Johnsrude I. S. (2011). Does semantic context benefit speech understanding through “top–down” processes? Evidence from time-resolved sparse fMRI. J. Cogn. Neurosci. 23, 3914–3932. doi: 10.1162/jocn_a_00084 21745006

[ref12] de Haan W. Mott K. van Straaten E. C. Scheltens P. Stam C. J. (2012). Activity dependent degeneration explains hub vulnerability in Alzheimer's disease. PLoS Comput. Biol. 8:e1002582. doi: 10.1371/journal.pcbi.1002582 22915996 PMC3420961

[ref13] de Leon M. J. DeSanti S. Zinkowski R. Mehta P. D. Pratico D. Segal S.. (2004). MRI and CSF studies in the early diagnosis of Alzheimer's disease. J. Intern. Med. 256, 205–223. doi: 10.1111/j.1365-2796.2004.01381.x 15324364

[ref14] Deal J. A. Betz J. Yaffe K. Harris T. Purchase-Helzner E. Satterfield S.. (2017). Hearing Impairment and incident dementia and cognitive decline in older adults: the health ABC study. J. Gerontol. A Biol. Sci. Med. Sci. 72, 703–709. doi: 10.1093/gerona/glw069 27071780 PMC5964742

[ref15] Del Sole A. Malaspina S. Magenta Biasina A. (2016). Magnetic resonance imaging and positron emission tomography in the diagnosis of neurodegenerative dementias. Funct. Neurol. 31, 205–215. doi: 10.11138/fneur/2016.31.4.205 28072381 PMC5231883

[ref16] Eckert M. A. Cute S. L. Vaden K. I. Jr. Kuchinsky S. E. Dubno J. R. (2012). Auditory cortex signs of age-related hearing loss. J. Assoc. Res. Otolaryngol. 13, 703–713. doi: 10.1007/s10162-012-0332-5 22618352 PMC3441956

[ref17] Eckert M. A. Vaden K. I. Jr. Dubno J. R. (2019). Age-related hearing loss associations with changes in brain morphology. Trends Hear. 23:2331216519857267. doi: 10.1177/2331216519857267 31213143 PMC6585256

[ref18] Ernst A. Frisen J. (2015). Adult neurogenesis in humans- common and unique traits in mammals. PLoS Biol. 13:e1002045. doi: 10.1371/journal.pbio.1002045 25621867 PMC4306487

[ref19] Ezzati A. Katz M. J. Zammit A. R. Lipton M. L. Zimmerman M. E. Sliwinski M. J.. (2016). Differential association of left and right hippocampal volumes with verbal episodic and spatial memory in older adults. Neuropsychologia 93, 380–385. doi: 10.1016/j.neuropsychologia.2016.08.016 27542320 PMC5154822

[ref20] Gates G. A. Mills J. H. (2005). Presbycusis. Lancet 366, 1111–1120. doi: 10.1016/S0140-6736(05)67423-5 16182900

[ref21] Giroud N. Lemke U. Reich P. Matthes K. L. Meyer M. (2017). The impact of hearing aids and age-related hearing loss on auditory plasticity across three months - An electrical neuroimaging study. Hear. Res. 353, 162–175. doi: 10.1016/j.heares.2017.06.012 28705608

[ref22] Gordon-Salant S. Fitzgibbons P. J. Friedman S. A. (2007). Recognition of time-compressed and natural speech with selective temporal enhancements by young and elderly listeners. J. Speech Lang. Hear. Res. 50, 1181–1193. doi: 10.1044/1092-4388(2007/082) 17905904

[ref23] Gould E. McEwen B. S. Tanapat P. Galea L. A. Fuchs E. (1997). Neurogenesis in the dentate gyrus of the adult tree shrew is regulated by psychosocial stress and NMDA receptor activation. J. Neurosci. 17, 2492–2498. doi: 10.1523/JNEUROSCI.17-07-02492.1997 9065509 PMC6573503

[ref24] Griffiths T. D. Lad M. Kumar S. Holmes E. McMurray B. Maguire E. A.. (2020). How can hearing loss cause dementia? Neuron 108, 401–412. doi: 10.1016/j.neuron.2020.08.003 32871106 PMC7664986

[ref25] Hederstierna C. Rosenhall U. (2016). Age-related hearing decline in individuals with and without occupational noise exposure. Noise Health 18, 21–25. doi: 10.4103/1463-1741.174375 26780958 PMC4918675

[ref26] Hollands C. Tobin M. K. Hsu M. Musaraca K. Yu T. S. Mishra R.. (2017). Depletion of adult neurogenesis exacerbates cognitive deficits in Alzheimer's disease by compromising hippocampal inhibition. Mol. Neurodegener. 12:64. doi: 10.1186/s13024-017-0207-7 28886753 PMC5591545

[ref27] Homans N. C. Metselaar R. M. Dingemanse J. G. van der Schroeff M. P. Brocaar M. P. Wieringa M. H.. (2017). Prevalence of age-related hearing loss, including sex differences, in older adults in a large cohort study. Laryngoscope 127, 725–730. doi: 10.1002/lary.26150 27377351

[ref28] Horgusluoglu-Moloch E. Risacher S. L. Crane P. K. Hibar D. Thompson P. M. Saykin A. J.. (2019). Genome-wide association analysis of hippocampal volume identifies enrichment of neurogenesis-related pathways. Sci. Rep. 9:14498. doi: 10.1038/s41598-019-50507-3 31601890 PMC6787090

[ref29] Houben S. Leroy K. Ando K. Yilmaz Z. Widomski C. Buee L.. (2019). Genetic ablation of tau in postnatal neurons rescues decreased adult hippocampal neurogenesis in a tauopathy model. Neurobiol. Dis. 127, 131–141. doi: 10.1016/j.nbd.2019.02.021 30818066

[ref30] Jack C. R. Jr. Dickson D. W. Parisi J. E. Xu Y. C. Cha R. H. O'Brien P. C.. (2002). Antemortem MRI findings correlate with hippocampal neuropathology in typical aging and dementia. Neurology 58, 750–757. doi: 10.1212/wnl.58.5.750 11889239 PMC2745935

[ref31] Kang Y. Na D. L. Hahn S. (1997). A validity study on the Korean Mini-Mental State Examination (K-MMSE) in dementia patients. J. Korean Neurol. Assoc. 15, 300–308.

[ref32] Kempermann G. Kuhn H. G. Gage F. H. (1997). More hippocampal neurons in adult mice living in an enriched environment. Nature 386, 493–495. doi: 10.1038/386493a0 9087407

[ref33] Komuro Y. Xu G. Bhaskar K. Lamb B. T. (2015). Human tau expression reduces adult neurogenesis in a mouse model of tauopathy. Neurobiol. Aging 36, 2034–2042. doi: 10.1016/j.neurobiolaging.2015.03.002 25863528 PMC4724414

[ref34] Kraus K. S. Mitra S. Jimenez Z. Hinduja S. Ding D. Jiang H.. (2010). Noise trauma impairs neurogenesis in the rat hippocampus. Neuroscience 167, 1216–1226. doi: 10.1016/j.neuroscience.2010.02.071 20206235 PMC2952397

[ref35] Kreisel T. Frank M. G. Licht T. Reshef R. Ben-Menachem-Zidon O. Baratta M. V.. (2014). Dynamic microglial alterations underlie stress-induced depressive-like behavior and suppressed neurogenesis. Mol. Psychiatry 19, 699–709. doi: 10.1038/mp.2013.155 24342992

[ref36] Kumar S. Gander P. E. Berger J. I. Billig A. J. Nourski K. V. Oya H.. (2021). Oscillatory correlates of auditory working memory examined with human electrocorticography. Neuropsychologia 150:107691. doi: 10.1016/j.neuropsychologia.2020.107691 33227284 PMC7884909

[ref37] Kumar S. Joseph S. Gander P. E. Barascud N. Halpern A. R. Griffiths T. D. (2016). A brain system for auditory working memory. J. Neurosci. 36, 4492–4505. doi: 10.1523/JNEUROSCI.4341-14.2016 27098693 PMC4837683

[ref38] Kurioka T. Mogi S. Yamashita T. (2021). Decreasing auditory input induces neurogenesis impairment in the hippocampus. Sci. Rep. 11:423. doi: 10.1038/s41598-020-80218-z 33432038 PMC7801596

[ref39] Lad M. Holmes E. Chu A. Griffiths T. D. (2020). Speech-in-noise detection is related to auditory working memory precision for frequency. Sci. Rep. 10:13997. doi: 10.1038/s41598-020-70952-9 32814792 PMC7438331

[ref40] Lazarov O. Hollands C. (2016). Hippocampal neurogenesis: Learning to remember. Prog. Neurobiol. 138-140, 1–18. doi: 10.1016/j.pneurobio.2015.12.006 26855369 PMC4828289

[ref41] Lemaire V. Koehl M. Le Moal M. Abrous D. N. (2000). Prenatal stress produces learning deficits associated with an inhibition of neurogenesis in the hippocampus. Proc. Natl. Acad. Sci. U. S. A. 97, 11032–11037. doi: 10.1073/pnas.97.20.11032 11005874 PMC27143

[ref42] Lewczuk P. Mroczko B. Fagan A. Kornhuber J. (2015). Biomarkers of Alzheimer's disease and mild cognitive impairment: a current perspective. Adv. Med. Sci. 60, 76–82. doi: 10.1016/j.advms.2014.11.002 25579841

[ref43] Liberman M. C. (2017). Noise-induced and age-related hearing loss: new perspectives and potential therapies. F1000Res 6:927. doi: 10.12688/f1000research.11310.1 28690836 PMC5482333

[ref44] Lin F. R. Ferrucci L. An Y. Goh J. O. Doshi J. Metter E. J.. (2014). Association of hearing impairment with brain volume changes in older adults. NeuroImage 90, 84–92. doi: 10.1016/j.neuroimage.2013.12.059 24412398 PMC3951583

[ref45] Lin F. R. Metter E. J. O'Brien R. J. Resnick S. M. Zonderman A. B. Ferrucci L. (2011). Hearing loss and incident dementia. Arch. Neurol. 68, 214–220. doi: 10.1001/archneurol.2010.362 21320988 PMC3277836

[ref46] Lin F. R. Yaffe K. Xia J. Xue Q. L. Harris T. B. Purchase-Helzner E.. (2013). Hearing loss and cognitive decline in older adults. JAMA Intern. Med. 173, 293–299. doi: 10.1001/jamainternmed.2013.1868 23337978 PMC3869227

[ref47] Liu L. Shen P. He T. Chang Y. Shi L. Tao S.. (2016). Noise induced hearing loss impairs spatial learning/memory and hippocampal neurogenesis in mice. Sci. Rep. 6:20374. doi: 10.1038/srep20374 26842803 PMC4740884

[ref48] Liu H. Zhang H. Ma Y. (2021). Molecular mechanisms of altered adult hippocampal neurogenesis in Alzheimer's disease. Mech. Ageing Dev. 195:111452. doi: 10.1016/j.mad.2021.111452 33556365

[ref49] Livingston G. Sommerlad A. Orgeta V. Costafreda S. G. Huntley J. Ames D.. (2017). Dementia prevention, intervention, and care. Lancet 390, 2673–2734. doi: 10.1016/S0140-6736(17)31363-6 28735855

[ref50] Maharani A. Dawes P. Nazroo J. Tampubolon G. Pendleton N. SENSE-Cog WP1 group (2018). Longitudinal relationship between hearing aid use and cognitive function in older Americans. J. Am. Geriatr. Soc. 66, 1130–1136. doi: 10.1111/jgs.15363 29637544

[ref51] McConathy J. Sheline Y. I. (2015). Imaging biomarkers associated with cognitive decline: a review. Biol. Psychiatry 77, 685–692. doi: 10.1016/j.biopsych.2014.08.024 25442005 PMC4362908

[ref52] Michelmann S. Price A. R. Aubrey B. Strauss C. K. Doyle W. K. Friedman D.. (2021). Moment-by-moment tracking of naturalistic learning and its underlying hippocampo-cortical interactions. Nat. Commun. 12:5394. doi: 10.1038/s41467-021-25376-y 34518520 PMC8438040

[ref53] Mu Y. Gage F. H. (2011). Adult hippocampal neurogenesis and its role in Alzheimer's disease. Mol. Neurodegener. 6:85. doi: 10.1186/1750-1326-6-85 22192775 PMC3261815

[ref54] O’Keefe J. Dostrovsky J. (1971). The hippocampus as a spatial map. Preliminary evidence from unit activity in the freely-moving rat. Brain Res. 34, 171–175. doi: 10.1016/0006-8993(71)90358-1 5124915

[ref56] Ouda L. Profant O. Syka J. (2015). Age-related changes in the central auditory system. Cell Tissue Res. 361, 337–358. doi: 10.1007/s00441-014-2107-2 25630878

[ref57] Pardoe H. Pell G. S. Abbott D. F. Berg A. T. Jackson G. D. (2008). Multi-site voxel-based morphometry: methods and a feasibility demonstration with childhood absence epilepsy. NeuroImage 42, 611–616. doi: 10.1016/j.neuroimage.2008.05.007 18585930 PMC2603188

[ref58] Park S. Y. Kim M. J. Kim H. L. Kim D. K. Yeo S. W. Park S. N. (2018). Cognitive decline and increased hippocampal p-tau expression in mice with hearing loss. Behav. Brain Res. 342, 19–26. doi: 10.1016/j.bbr.2018.01.003 29317248

[ref59] Peelle J. E. Troiani V. Grossman M. Wingfield A. (2011). Hearing loss in older adults affects neural systems supporting speech comprehension. J. Neurosci. 31, 12638–12643. doi: 10.1523/JNEUROSCI.2559-11.2011 21880924 PMC3175595

[ref60] Pichora-Fuller M. K. Schneider B. A. Macdonald E. Pass H. E. Brown S. (2007). Temporal jitter disrupts speech intelligibility: a simulation of auditory aging. Hear. Res. 223, 114–121. doi: 10.1016/j.heares.2006.10.009 17157462

[ref61] Pristera A. Saraulli D. Farioli-Vecchioli S. Strimpakos G. Costanzi M. di Certo M. G.. (2013). Impact of N-tau on adult hippocampal neurogenesis, anxiety, and memory. Neurobiol. Aging 34, 2551–2563. doi: 10.1016/j.neurobiolaging.2013.05.010 23769395

[ref62] Profant O. Balogova Z. Dezortova M. Wagnerova D. Hajek M. Syka J. (2013). Metabolic changes in the auditory cortex in presbycusis demonstrated by MR spectroscopy. Exp. Gerontol. 48, 795–800. doi: 10.1016/j.exger.2013.04.012 23648586

[ref63] Profant O. Skoch A. Balogova Z. Tintera J. Hlinka J. Syka J. (2014). Diffusion tensor imaging and MR morphometry of the central auditory pathway and auditory cortex in aging. Neuroscience 260, 87–97. doi: 10.1016/j.neuroscience.2013.12.010 24333969

[ref64] Profant O. Skoch A. Tintera J. Svobodova V. Kucharova D. Svobodova Burianova J.. (2020). The influence of aging, hearing, and tinnitus on the morphology of cortical gray matter, amygdala, and hippocampus. Front. Aging Neurosci. 12:553461. doi: 10.3389/fnagi.2020.553461 33343328 PMC7746808

[ref65] Profant O. Tintera J. Balogova Z. Ibrahim I. Jilek M. Syka J. (2015). Functional changes in the human auditory cortex in ageing. PLoS One 10:e0116692. doi: 10.1371/journal.pone.0116692 25734519 PMC4348517

[ref66] Ren F. Ma W. Li M. Sun H. Xin Q. Zong W.. (2018). Gray matter atrophy is associated with cognitive impairment in patients with presbycusis: a comprehensive morphometric study. Front. Neurosci. 12:744. doi: 10.3389/fnins.2018.00744 30405333 PMC6205975

[ref67] Rigters S. C. Bos D. Metselaar M. Roshchupkin G. V. Baatenburg de Jong R. J. Ikram M. A.. (2017). Hearing impairment is associated with smaller brain volume in aging. Front. Aging Neurosci. 9:2. doi: 10.3389/fnagi.2017.00002 28163683 PMC5247429

[ref68] Roth T. N. Hanebuth D. Probst R. (2011). Prevalence of age-related hearing loss in Europe: a review. Eur. Arch. Otorhinolaryngol. 268, 1101–1107. doi: 10.1007/s00405-011-1597-8 21499871 PMC3132411

[ref69] Schloesser R. J. Jimenez D. V. Hardy N. F. Paredes D. Catlow B. J. Manji H. K.. (2014). Atrophy of pyramidal neurons and increased stress-induced glutamate levels in CA3 following chronic suppression of adult neurogenesis. Brain Struct. Funct. 219, 1139–1148. doi: 10.1007/s00429-013-0532-8 23483239 PMC3795860

[ref70] Schoenfeld T. J. McCausland H. C. Morris H. D. Padmanaban V. Cameron H. A. (2017). Stress and loss of adult neurogenesis differentially reduce hippocampal volume. Biol. Psychiatry 82, 914–923. doi: 10.1016/j.biopsych.2017.05.013 28629541 PMC5683934

[ref71] Shi F. Liu B. Zhou Y. Yu C. Jiang T. (2009). Hippocampal volume and asymmetry in mild cognitive impairment and Alzheimer's disease: Meta-analyses of MRI studies. Hippocampus 19, 1055–1064. doi: 10.1002/hipo.20573 19309039

[ref72] Shukla M. Roy K. Kaur C. Nayak D. Mani K. V. Shukla S.. (2019). Attenuation of adverse effects of noise induced hearing loss on adult neurogenesis and memory in rats by intervention with Adenosine A2A receptor agonist. Brain Res. Bull. 147, 47–57. doi: 10.1016/j.brainresbull.2019.02.006 30771409

[ref73] Tun P. A. O'Kane G. Wingfield A. (2002). Distraction by competing speech in young and older adult listeners. Psychol. Aging 17, 453–467. doi: 10.1037//0882-7974.17.3.453 12243387

[ref74] Uchida Y. Nishita Y. Kato T. Iwata K. Sugiura S. Suzuki H.. (2018). Smaller Hippocampal Volume and Degraded Peripheral Hearing Among Japanese Community Dwellers. Front. Aging Neurosci. 10:319. doi: 10.3389/fnagi.2018.00319 30386230 PMC6198789

[ref75] van Praag H. Kempermann G. Gage F. H. (2000). Neural consequences of environmental enrichment. Nat. Rev. Neurosci. 1, 191–198. doi: 10.1038/35044558 11257907

[ref76] Voss M. W. Soto C. Yoo S. Sodoma M. Vivar C. van Praag H. (2019). Exercise and Hippocampal Memory Systems. Trends Cogn. Sci. 23, 318–333. doi: 10.1016/j.tics.2019.01.006 30777641 PMC6422697

[ref55] World Health Organization (1991). Report of the informal working group on prevention of deafness and hearing impairment Programme planning, Geneva, 18–21 June 1991.

[ref77] Yang C. H. Schrepfer T. Schacht J. (2015). Age-related hearing impairment and the triad of acquired hearing loss. Front. Cell. Neurosci. 9:276. doi: 10.3389/fncel.2015.00276 26283913 PMC4515558

